# Management of Horizontal Root Fracture in Anterior Teeth: A Case Report

**DOI:** 10.7759/cureus.29402

**Published:** 2022-09-21

**Authors:** Nishi Malviya, Nilima R Thosar, Rutuja Wayakar, Monika Khubchandani, Akansha V Bansod

**Affiliations:** 1 Pedodontics and Preventive Dentistry, Sharad Pawar Dental College and Hospital, Datta Meghe Institute of Medical Sciences, Wardha, IND; 2 Pediatric and Preventive Dentistry, Sharad Pawar Dental College and Hospital, Datta Meghe Institute of Medical Sciences, Wardha, IND; 3 Pediatric Dentistry, Sharad Pawar Dental College and Hospital, Datta Meghe Institute of Medical Sciences, Wardha, IND; 4 Prosthodontics, Sharad Pawar Dental College and Hospital, Datta Meghe Institute of Medical Sciences, Wardha, IND

**Keywords:** thermoplastized gutta percha., traumatic dental injuries (tdi’s), mineral trioxide aggregrate, horizontal root fracture, immature root apex

## Abstract

Following traumatic injury, pulpal and periapical pathosis in an immature anterior tooth is common, and treating open apices in these situations is a persistent problem for pedodontists. This is because there is no apical constriction, which would prevent the obturated material from forming an excellent three-dimensional seal or adaption within the canal system. Mineral Trioxide Aggregate (MTA) offers a good choice when employed to create an apical barrier. In this case report, an open apex and periapical lesion involving maxillary right central incisor #21 with the MTA are shown with a six-week follow-up result after being treated for four weeks with triple antibiotic paste as an intra-canal medication. The successful healing of tooth 21 and the diminution of the periapical radiolucency at one-week follow-up were observed.

## Introduction

Children between the ages of 8 and 12 most frequently get traumatic dental injuries (TDIs). These wounds could cause pulpal necrosis, inhibiting root growth and leaving a developing root apex [1]. Open apices and weaker dentinal walls make it difficult to treat effectively from an endodontic and restorative perspective, which frequently predisposes to root fractures, especially in the cervical region [2]. TDI prevalences ranging from 2.4% to 58.6% [3].

TDIs are the most common reasons for needing emergency dental care and typically originate from unfavorable events such as falls, car accidents, impact sports, and fights. The high force causes horizontal root breakages, identified by fractures of the complex root structures in the coronal, mid, or apical regions. Fractures are linked to damage to the periodontal ligament, the supporting alveolar bone, and tooth separation. They can involve pulp, dentin, and cement. The coronal part of the tooth is often displaced, while the apical segment is rarely involved [4].

A systematic approach with clinical and radiographical examinations is essential in diagnosing injuries to the teeth. The most frequently encountered scenario is luxation damage to the coronal fragment. A horizontal or radiolucent line separating the displaced coronal component from the apical one can be seen on radiographic inspection, which validates the diagnosis [5].

The location and pulp viability of a root fracture determine its clinical care. Conservative treatment involves realigning the teeth, immobilization, and occlusion alleviation when the coronal fragment is displaced. According to reports, satisfactory treatment outcomes were attained in up to 80% of instances. The best results come from immediate immobilization within an hour of the event. If the fracture fragment is nonvital or pathological symptoms arise during the follow-up period, endodontic treatment should be performed through the apical end of the coronal portion. Intra-radicular splinting and associated restorative care may be used as an additional dental treatment [6].

## Case presentation

A young ten-year-old girl reported to the outpatient department in the Department of Pediatric and Preventive Dentistry with the chief complaint of pain, swelling, and poor esthetics in the upper front region of the jaw for 15 days.

The patient was apparently alright 15 days back then; she experienced pain with 21, which was dull aching, with no specific aggravating and relieving factors, and swelling in the gingival region with 21 was gradual in onset. The patient gives a history of trauma one month back due to a fall while playing. History of mobility of tooth for 15 days was also reported. No history of loss of consciousness, dizziness, and nose bleeds. 

**Figure 1:**
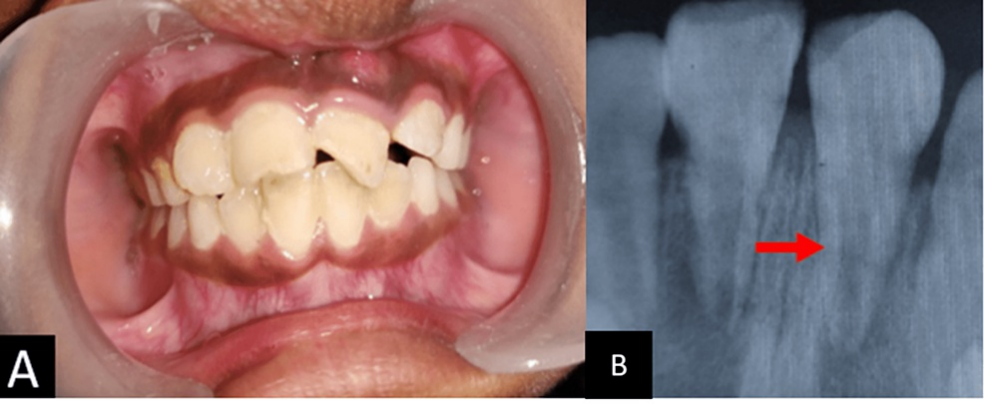
Preoperative images: A: Clinical B: Radiograph In the clinical Figure 1A, Ellis Class I fracture was seen with the right central incisor, and Ellis Class III fracture with the left central incisor. In Figure 1B, periapical rarefaction with a fracture in the middle third root region and periodontal ligament widening was noted in 21.

On preoperative examination, Ellis class I fracture was noted at 11, and Ellis class III fracture was noted at 21. On radiographic examination, the middle one-third root fracture was noted with 21 with periapical rarefaction in 21 as seen in Figure 1.

Treatment

**Figure 2:**
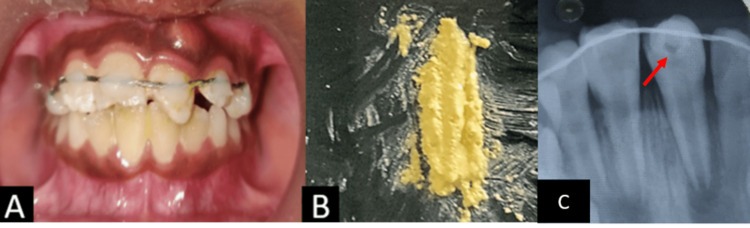
Immediate post-op photograph A: Splinting seen clinically, B: Triple antibiotic paste, C: Radiographic image- Triple antibiotic pate placement and splinting

The treatment plan was formulated in order to stabilize the mobile fragment with a ligature wire splint (Dentomech MBT Orthodontic Ligature Wire, Davangere, Karnataka, India) for four weeks. Wire splitting was bonded with flowable restorative composite (3M Espe Filtek supreme flowable), and the splints were placed by assessing the correct position of the tooth radiographically and clinically with 53,12,11,21,22,63. After the placement of composite wire splints, access opening was done in 21, followed by application of the triple antibiotic paste (Ciprofloxacin 100mg, metronidazole 500mg, and minocycline 100mg in the ratio 1:1:1) and restored with glass ionomer cement (GC Gold Label 9 Posterior Restorative). In the radiographic image, we can appreciate the radiopaque wiring in the coronal aspect of the involved teeth 12,11,21,22. A periapical lesion with open apex and periodontal ligament widening can also be appreciated with 21, as seen in Figure 2.

Follow-up was done for four weeks, and every week clinical and radiographic assessment was done. The patient was kept on follow-up every week, and clinical and radiographic assessment was done periodically.

**Figure 3:**
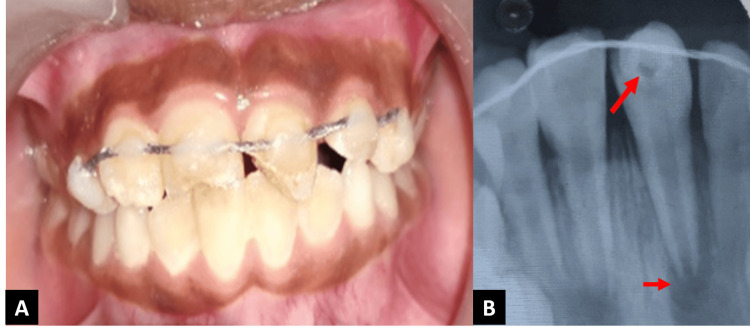
At first follow-up visit A: Clinical, B: Radiograph Clinically, regression of swelling was seen after one week and there were no significant changes seen radiographically (red arrow).

In the first follow-up visit, clinically composite wire splinting was seen with 53,12,11,21,22,63, and there was an absence of the gingival swelling, which was previously seen when the patient reported, and radiographically there were no changes observed as seen in Figure 3.

**Figure 4:**
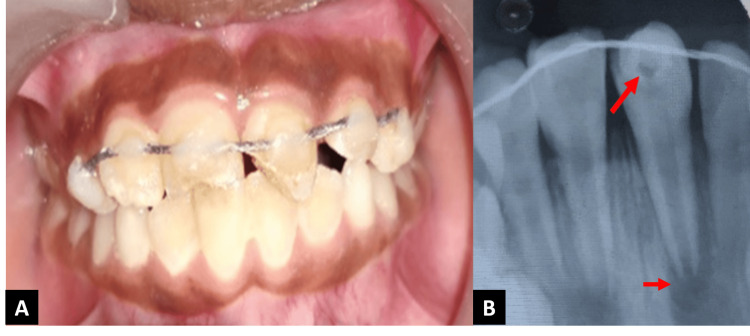
Second follow-up A: Clinical composite wire splinting was done (Flexible splints), B: Radiograph Clinically, a complete absence of gingival swelling and the absence of a periapical lesion was observed radiographically (red arrow).

Second-week follow-up: clinical and radiographic follow-up of the patient was made. Clinically, a complete absence of gingival swelling and the absence of a periapical lesion was observed radiographically, as seen in Figure 4.

**Figure 5:**
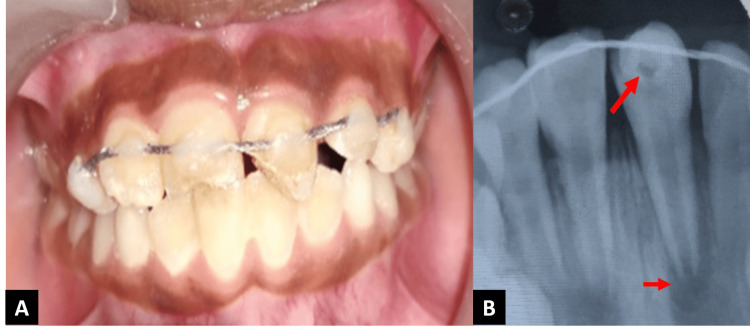
Third Follow-up A: Clinical, B: Radiograph In the third follow-up, clinically splinting was seen intact as done, and radiographically, a reduction in the size of periapical rarefaction was observed, and periodontal ligament widening was absent, which suggests healing (red arrow).

In the third follow-up, clinically splinting was seen intact as done, and radiographically, a reduction in the size of periapical rarefaction was observed, and periodontal ligament widening was absent, which suggests healing, as seen in Figure 5.

**Figure 6:**
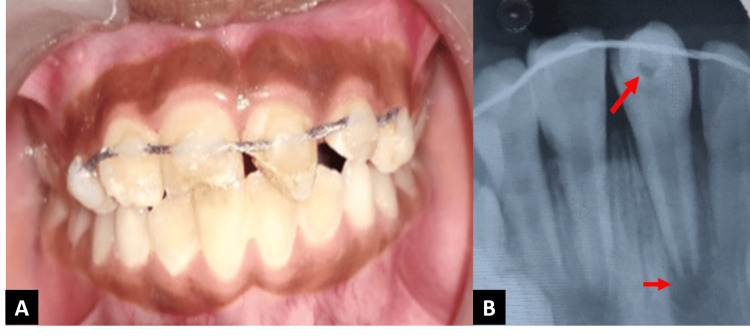
Fourth Follow-up A: Clinically flexible splint was visible, B: Radiographically significant reduction of the periapical lesion was appreciated

In the fourth-week follow-up, clinically splinting was observed, and radiographically periapical healing was observed, as seen in Figure 6 (red arrow).

**Figure 7:**
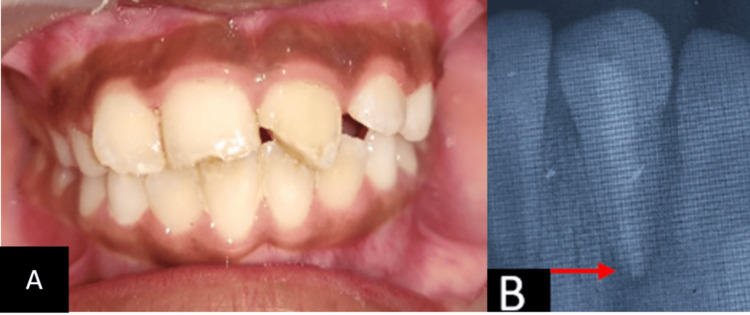
Fifth Follow-up A: Clinical, B: Radiograph After four weeks splinting was removed. Mobility of the tooth was reduced significantly from Glickman's Grade II to Grade I mobility. Also, the periapical lesion was significantly reduced from approximately 3*4mm to 1*1mm, and triple antibiotic paste was replaced with mineral trioxide aggregate (MTA) was placed.

Fifth appointment: After four weeks splinting was removed. Mobility of the tooth was reduced significantly from Glickman's Grade II to Grade I mobility. Hence, splinting was removed. Also, the periapical lesion was significantly reduced from approximately 3*4mm to 1*1mm, and triple antibiotic paste was replaced with mineral trioxide aggregate (MTA) (Kids e Dental LLP B127) placed as seen in Figure 7.

**Figure 8:**
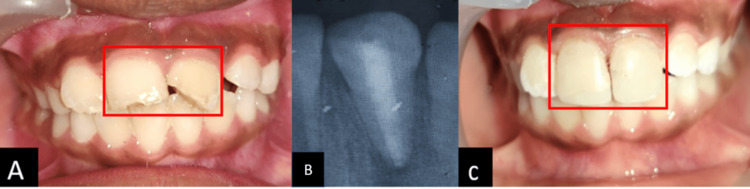
Sixth Follow-up visit A: Clinical, B: Radiograph post obturation with thermoplasticized Gutta-percha, C: Composite restoration After setting of MTA, obturation was done with thermoplastic gutta-percha (Dentsply Calamus Dual Obturation System Kit) with 21, followed by composite restoration (3m Espe Filtek Bulk Fill Composite Syringe) with 21.

Sixth visit: After setting of MTA, obturation was done with thermoplastic gutta-percha (Dentsply Calamus Dual Obturation System Kit) with 21, followed by composite restoration (3m Espe Filtek Bulk Fill Composite Syringe) with 21 (Figure 8).

## Discussion

Open apices have thin, delicate dentinal walls that are prone to fracture because of their nature. It is mainly impossible to cleanse the canals and necrotic material from the root canal system. When handling teeth with open apices, the practitioner faces a significant problem due to the greater possibility of overextension of materials into the periapex and challenges with cleaning and shape. One way to obturate juvenile teeth with root canal filling material without causing apical closure is to establish appropriate root end closure with MTA or Biodentine.

A difficult task for clinicians has always been placing intracanal medication in cases of persistent or significant periapical pathosis. Several antibiotic combinations, particularly metronidazole, minocycline, and ciprofloxacin [usually referred to as a Triple antibiotic mixture] (TAP), are highly effective in eliminating endodontic pathological microbiota both in-situ and in-vitro [7].

The complex microbial flora found in the canal system, which often includes both aerobic and anaerobic species, makes it unlikely that administering a single antibiotic will completely eradicate bacteria from the canal. The “Niigata University School of Dentistry's Cariology Research Unit” developed the lesion sterilization and tissue repair (LSTR) concept to address these drawbacks [8]. This uses ciprofloxacin, metronidazole, and minocycline as three different antibiotics to disinfect. The use of TAP for traumatized teeth presenting with periapical pathosis has been shown to have various positive effects in the past, according to published literature [9]. In the case study from 2005, Ozan and Er et al. [10] concluded that combining combination antibiotic medications in the triple antibiotic paste was an alternative for resolving large cyst-like lesions. 

According to the International Association of Dental Traumatology (IADT) 2020, flexible splints are recommended for root fractures, avulsions, and luxations. Splinting of the teeth may be performed for bone segment immobilization in the event of alveolar bone fractures. When employing wire-composite splints, physiological stabilization can be achieved with stainless steel wire up to 0.4 mm in diameter. To keep the repositioned tooth in its proper position, promote initial healing, and provide comfort and controlled function, splinting is seen to be the best practice. To prevent plaque buildup and subsequent infection, it is crucial to keep composite and bonding chemicals away from the gingiva and proximal areas. As a result, the marginal gingiva and bone can repair more effectively. The splint length will vary depending on the kind of injury.

In teeth with necrotic pulp and open apices, the use of MTA was advised. The suggestions for developing the MTA apical plug were based on the material's beneficial traits, such as its good biocompatibility and lower cytotoxicity due to its higher alkalinity. The attraction of blastic cells by calcium and phosphate ions promotes the repair of periapical tissues to a normal state. It creates a favorable environment for bone deposition without inducing an inflammatory response. Additionally, a new cementum formation was said to surround MTA material [11].

As the resilon exhibits a thermoplasticity similar to gutta-percha and a similar ability to seal the lateral ducts and depressions present in the root canals, according to the majority of the published evidence, thermoplastic gutta-percha was used as an obturating material through injection technique. In reality, the material's ability of thermoplasticity determines how efficiently the vertical condensation occurs. They are, therefore, entirely appropriate for this kind of method. Compared to other materials, the resilon showed a superior ability to close or seal the lateral ducts, particularly in the apical portion of the root [12].

## Conclusions

An open apex and pulpal necrosis are frequently the results of damage to the anterior teeth. Following trauma, managing anterior teeth becomes a serious issue and a real struggle. Immediately, high-quality care is always required because of patients' high aesthetic expectations. It may be beneficial to completely seal off the root canal system and fortify developing teeth with bioactive materials like Biodentine/MTA. Therefore, the MTA material can be recommended for the creation of the calcific barrier or an apical seal. In the above-mentioned case, MTA apexification was performed successfully as well as esthetic restoration keeping in mind the patients' demand for esthetics. 
